# Implementing an Activity Tracker to Increase Motivation for Physical Activity in Patients With Diabetes in Primary Care: Strengths, Weaknesses, Opportunities and Threats (SWOT) Analysis

**DOI:** 10.2196/44254

**Published:** 2023-03-10

**Authors:** Cynthia Pelletier, Christian Chabot, Marie-Pierre Gagnon, Caroline Rhéaume

**Affiliations:** 1 Department of Family Medicine and Emergency Medicine Faculty of Medicine Université Laval Québec, QC Canada; 2 VITAM – Centre de recherche en santé durable Université Laval Québec, QC Canada; 3 Faculty of Nursing Université Laval Québec, QC Canada; 4 Centre de recherche de l’Institut universitaire de cardiologie et de pneumologie de Québec Québec, QC Canada

**Keywords:** activity tracker, type 2 diabetes, family medicine research, SWOT analysis, physical activity, physical activity motivation, diabetes, implementation, chronic disease, intervention, questionnaire, tool

## Abstract

**Background:**

Many projects related to technology implementation in the context of chronic diseases have been developed over the years to better manage lifestyle medicine interventions and improve patient care. However, technology implementation in primary care settings remains challenging.

**Objective:**

The aim is to carry out a strengths, weaknesses, opportunities, and threats (SWOT) analysis (1) to assess satisfaction among patients with type 2 diabetes using an activity tracker to increase motivation for physical activity (PA) and (2) to explore the research and health care team’s perceptions of this technology’s implementation in a primary care setting.

**Methods:**

A 3-month hybrid type 1 study, which included 2 stages, was conducted in an academic primary health center in Quebec City, Quebec, Canada. In stage 1, a total of 30 patients with type 2 diabetes were randomized to the intervention (activity tracker) group or the control group. In stage 2, a SWOT analysis was performed on both patients and health care professionals to determine the components of successful technology implementation. Two questionnaires were used to gather feedback: a satisfaction and acceptability questionnaire concerning an activity tracker (15 patients in the intervention group) and a questionnaire based on the SWOT elements (15 patients in the intervention group and 7 health care professionals). Both questionnaires contained quantitative and qualitative questions. Qualitative variables from open questions were synthesized in a matrix and ranked according to apparition frequency and global importance. A thematic analysis was performed by the first author and validated by 2 coauthors separately. The information gathered was triangulated to propose recommendations that were then approved by the team. Both quantitative (randomized controlled trial participants) and qualitative (randomized controlled trial participants and team) results were combined for recommendations.

**Results:**

In total, 86% (12/14) of the participants were satisfied with their activity tracker use and 75% (9/12) felt that it incited them to stick to their PA program. The main strengths of the team members’ perspectives were the project initiation and involvement of a patient partner, the study design, the team, and the device. The weaknesses were the budgetary constraints, the turnover, and the technical issues. The opportunities were the primary care setting, the loan of equipment, and common technology. The threats were recruitment issues, administrative challenges, technological difficulties, and a single research site.

**Conclusions:**

Patients with type 2 diabetes were satisfied with their activity tracker used to improve motivation for PA. Health care team members agreed that implementation can be done in primary care, but some challenges remain in using this technological tool in clinical practice regularly.

**Trial Registration:**

ClinicalTrials.gov NCT03709966; https://clinicaltrials.gov/ct2/show/NCT03709966

## Introduction

### Background

Over the years, many research projects related to technology implementation in the context of chronic diseases have been developed in primary care. One of the main effects is to contribute to a favorable lifestyle medicine change, which may have positive repercussions on patient health and the management of chronic diseases [1-4].

Recently, a scoping review by Clarkson et al [5] identified the increased use of digital tools, in combination with human support, to help people with long-term conditions and to maintain physical activity (PA). This review shows that web-based digital tools continue to predominate with the more recent emergence of gamification, applications, and virtual environments. However, most participants were from younger age groups, the use and description of the theory in the development of the tools were limited, and most studies highlighted the need for human engagement to support their use [5]. The lack of digital tools for multimorbid long-term conditions, longer-term follow-ups, understanding participants’ experiences, and informing future questions about the effectiveness were the obvious gaps [5].

Another scoping review, conducted by Motahari-Nezhad et al [6], revealed that clinical evidence concerning digital biomarkers has been systematically reviewed across a wide range of study populations, interventions, digital devices, and sensor technologies with the dominance of PA and cardiac monitors. To understand the clinical value of digital biomarkers, the strength and quality of the evidence on their health consequences need to be systematically evaluated.

Indeed, the use of digital technology to help patients with chronic diseases such as type 2 diabetes is an emerging field of research. There is a variety of equipment available to patients, including consumer activity trackers, pedometers, smartphone apps, and blood glucose monitors. The advantages of these technologies are that they can allow health care professionals to remotely monitor patients and reduce the need for patients to regularly attend clinics [7]. Recently, a mixed methods study by Hodgson et al [4] explored the use of an activity tracker for 4 weeks to support an active lifestyle in adults diagnosed with type 2 diabetes. Overall, the results demonstrated that fitness trackers could support an active lifestyle in adults with type 2 diabetes, but more detailed discussions with health care professionals could identify methods of integrating activity trackers into patient care [4]. Additionally, a multicenter prospective observational study (set up for 7 weeks) has evaluated the feasibility of using a nonmedicalized device to monitor the lifestyle of elderly patients with type 2 diabetes in a primary care setting [8]. Researchers observed a high level of acceptance of portable devices based on the impressions of patients and health care professionals [8]. Nevertheless, the authors suggest further studies to assess devices’ acceptability for longer periods [8].

Additionally, it is important to point out that technical failures were faced in many studies, such as log-in difficulties [3], connectivity errors [3,9], a lack of reliability and validity [3,9,10], as well as some patients feeling overwhelmed by the technical complexities of the activity tracker and its software [4,10]. Patient motivation dropped when such complications occurred [3]. The limited resources, especially in terms of staff [9,11] and money [9], were also reported as barriers to implementation.

### Context of the Study

A hybrid type 1 trial aims to determine the effectiveness of clinical intervention and to better understand the implementation context [12]. It is useful to explore if a clinical intervention works in a specific context and to gather potential barriers or facilitators to an intervention’s widespread implementation. Based on this design, a randomized pilot trial was conducted to evaluate the impact of an activity tracker on PA and cardiometabolic variables in a real-life settings among patients with type 2 diabetes [13,14]. Alongside this randomized pilot trial, we tested its effects on relevant outcomes while observing and gathering information from patients and the health care team with a strengths, weaknesses, opportunities, and threats (SWOT) analysis [15]. SWOT analysis has been used to determine the components of successful technology implementation in primary care settings, and its objective is to use the knowledge an organization has about its environment to formulate its strategy accordingly [15]. To the best of our knowledge, no study has simultaneously studied the feasibility and implementation among patients with type 2 diabetes using an activity tracker to increase motivation for PA in primary care with a SWOT analysis.

### Objectives

The aim of this study is to carry out a SWOT analysis to assess the acceptance of this novel technology by (1) assessing satisfaction among patients with type 2 diabetes using an activity tracker to increase motivation for PA and (2) exploring the health care team’s perception of its implementation in a primary care setting.

## Methods

### Hybrid Type 1 Study

We conducted a hybrid type 1 study for 3 months in an academic primary health center in Quebec City, Quebec, Canada. The study consisted of 2 stages. The first stage was a pilot randomized controlled trial to test a clinical intervention, and the second stage was to gather information on its implementation potential with patients’ and the team’s feedback using a SWOT analysis.

### First Stage: a Pilot Randomized Controlled Trial

The first component consisted of a pilot randomized controlled trial of 30 patients with 2 groups of 15 patients each to evaluate the impact of an activity tracker on PA and cardiometabolic variables in a real-life context among patients with type 2 diabetes. The complete study protocol was published on ClinicalTrials.gov (NCT03709966) [13]. The results of this study are published elsewhere [14]. The control group received routine follow-up, which included a PA promotion intervention supported by a kinesiologist. The intervention group is routinely followed up with the addition of an activity tracker (Fitbit Charge HR, Fitbit Inc) worn during the study. Participants in the intervention group were also provided with a tablet (iPad, Apple Inc) and with an application linked to the activity tracker (the Fitbit app). Cardiometabolic risk variables, PA motivation, and PA were assessed at the baseline and at the end of the trial.

### Second Stage: Gathering Information on Implementation Through a SWOT Analysis

The second component of the study consisted of gathering information on the delivery and potential of implementation. Feedback from patients and the health care team was gathered to perform a SWOT analysis. A SWOT analysis was particularly relevant in the context of this project in order to identify factors that could influence the replication of this intervention in a similar context. The SWOT analysis considers factors that are internal (strengths and weaknesses) and external (opportunities and threats) to the intervention. Thus, it can inform on both the design of an intervention and the strategic planning of its implementation [15].

### Satisfaction and Acceptability of the Activity Tracker: Patients’ Feedback

Satisfaction and acceptability of the activity tracker as well as the application were measured by a questionnaire inspired by the Technology Acceptance Model [16] and the System Usability Scale [17,18]. The 15 participants in the intervention group were invited at the end of the study to fill out the 10-question questionnaire on their satisfaction with the device and the support provided by the research team, their opinion about the information the device is providing, the impact on their lives, and their comments on the study. The questionnaire was in French and contained both quantitative and qualitative elements. The questionnaire is available in Multimedia Appendix 1.

### Implementation Questionnaire: Health Care Team’s Feedback

Research team members and health care professionals who were engaged in this project were also consulted. We used LimeSurvey, a web-based survey tool, to collect the team’s feedback [19]. In total, 12 participants and team members were invited to fill out an original questionnaire based on the SWOT analysis elements [15]. It contained 11 questions about their identification, the study’s strengths and weaknesses, the achievement of its objectives, the barriers faced, the facilitating factors, the integration of an activity tracker in primary care, and possible improvements. The questionnaire was in French and contained both quantitative and qualitative elements. A link to fill out the questionnaire was sent via email, and a reminder was also emailed a month later. The questionnaire is available in Multimedia Appendix 2.

### Ethics Approval

The authors are accountable for all aspects of the work. The trial was conducted in accordance with the Declaration of Helsinki (as revised in 2013) [20]. The study was approved by the institutional ethics board of the Centre intégré universitaire en santé et services sociaux de la Capitale-Nationale (No: 2017-2018-07), and informed consent was obtained from all the participants. Data were deidentified to preserve confidentiality. Furthermore, the data from the activity tracker are subject to the Fitbit privacy policy [21]. No financial compensation was given, but parking tickets for the clinic were provided.

### Data Analyses

Quantitative variables from selected answers (satisfaction and acceptability of the activity tracker’s questionnaire) were reported in frequency tables. Qualitative variables from open questions (the implementation questionnaire) were compiled in a file, and key concepts were identified by hand by the first author (CP). The first author then manually performed an inductive thematic analysis [22] to regroup key concepts as they emerged from data from both questionnaires, which were validated separately by 2 other coauthors (CR and MPG). The themes were then synthesized in a matrix and ranked according to apparition frequency and global importance. The information gathered was triangulated using the SWOT model (Opportunities-Strengths [O-S], Opportunities-Weaknesses [O-W], Threats-Strengths [T-S], and Threats-Weaknesses [T-W]) to propose recommendations that were then approved by the research team. For quantitative variables, normality was assessed using the Kolmogorov-Smirnov test. Variables following a normal distribution were expressed as mean (SD) values; otherwise, median (IQR) values were used. Missing data were excluded. The analyses were performed with the software SAS (version 9.4; SAS Institute).

## Results

### First Stage: Summary of Meaningful Results of the Randomized Pilot Trial Previously Published

The first stage of the study, the randomized pilot trial, showed that PA assessed by questionnaire increased in the group with a PA intervention supported by a kinesiologist (the control) and in the group with an activity tracker in addition to the PA intervention (the intervention). High-density lipoprotein cholesterol increased in the intervention group and decreased in the control group (*P*=.01). Glycated hemoglobin tended to decrease in both groups (*P*=.08). The full results are published elsewhere [14].

### Second Stage: Implementation Questionnaire and SWOT Analysis

#### Satisfaction and Acceptability Outcomes: Patients’ Feedback

The baseline demographics of participants in the intervention group are presented in Table 1. Since there was 1 dropout in the intervention group, 14 participants out of 15 completed the satisfaction and acceptability questionnaire. Satisfaction with the activity tracker use and technical support provided by the team are shown in Table 2. In total, 86% (12/14) of the participants were satisfied using their activity tracker. Some of the participants (11/14, 79%) were satisfied with the technical support provided by the team. The perceived usefulness of the information displayed by both the activity tracker and the application is reported in Table 3. Approximately 79% (11/14) of the participants found the information useful. The step count was perceived as the most useful parameter to track PA and for PA motivation (Table 4). More than half of the participants (6/11, 55%) were planning to buy an activity tracker after the study, 36% (4/11) were not planning to buy an activity tracker, and 9% (1/11) were undecided.

Participants were also asked to name the principal change they made to their lifestyle habits during the study. Many of them reported an increase in their PA, such as using the stairs, walking more, and doing more PA overall.

Participants were also asked if they continued to integrate PA into their daily routine once the study was over and why. Most of them said yes (n=12) and mentioned reasons such as pleasure, habit, feeling good, and achieving goals. If they answered yes, participants were asked to what extent the activity tracker incited them in sticking to their PA program once the study was completed. The results are presented in Table 5. In total, 75% (9/12) of the respondents thought the activity tracker had encouraged them to stick to their PA program upon completion of the study. One participant wrote: “It’s more realistic, I see that I can control my physical activity despite my schedule.”

**Table 1:** Baseline demographics of participants in the intervention group from the pilot randomized trial [14].

Baseline demographics		Intervention group
Participants, n		14
**Gender, n**		
	Male	9
	Female	5
Age (years), mean (SD)		62.1 (12.4)

**Table 2:** Satisfaction of the activity tracker use and technical support provided by the team (N=14).

Satisfaction	Satisfaction of the activity tracker use, n (%)	Satisfaction of the technical support, n (%)
Not at all satisfied	0 (0)	0 (0)
Not very satisfied	0 (0)	0 (0)
Somewhat satisfied	2 (14)	3 (21)
Satisfied	7 (50)	6 (43)
Very satisfied	5 (36)	5 (36)

**Table 3:** Usefulness of the information displayed by the activity tracker and the application (N=14).

Usefulness	Values, n (%)
Not at all useful	0 (0)
Not very useful	1 (7)
Somewhat useful	2 (14)
Useful	3 (22)
Very useful	8 (57)

**Table 4:** Most useful parameters to track physical activity and for physical activity motivation.

Parameter	Most useful parameters to track PA^a^ (n=17), n (%)	Most useful parameters for PA motivation (n=15), n (%)
Weight	3 (18)	4 (27)
Distance traveled	4 (23)	3 (20)
Step count	6 (35)	5 (33)
Minutes of being sedentary, mild, moderate, and strenuous PA	3 (18)	2 (13)
Calories burned	1 (6)	1 (7)

^a^PA: physical activity.

**Table 5:** Extent to which the activity tracker incited oneself to stick to the physical activity program once the study was over.

Incitation	Values (N=12), n (%)
Not incited at all	1 (8)
Not very incited	1 (8)
Somewhat incited	1 (8)
Incited	6 (50)
Very incited	3 (25)

#### Health Care Team’s Feedback

The implementation questionnaire was completed by 7 health care team members. There were 2 men and 5 women, and the mean age was 44.7 (SD 18.6) years. The health care team’s responders were composed of a family physician, researchers, a graduate student (MD-MSc), a professional coordinator, a kinesiologist, and a layperson (patient partner). Team members’ perceptions regarding the strengths, weaknesses, opportunities, and threats related to the implementation of an activity tracker to increase motivation for PA among patients with type 2 diabetes as well as patients’ feedback are summarized in a SWOT matrix (Textbox 1).

**SWOT (strengths, weaknesses, opportunities, and threats) matrix.**

**Strengths**

- Project initiation and involvement of a layperson
- Links to clinical interests
- Interprofessional collaboration or multidisciplinary team
- Accessibility and leadership of the principal investigator
- High satisfaction from participants, collaboration, and a desire to improve knowledge
- Training of research students
- Simple, available, and affordable tool
- Follow-up with the kinesiologist or phone call
- Objective follow-up of physical activity or motivational tool
- Small number of appointments
- Easy-to-fill questionnaires
- Precise measures of body composition

**Weaknesses**

- Tight budget: a few pedometers and activity trackers
- Small sample size: limited power and significance
- Part-time research coordinator: lack of follow-up measures and missing data
- Rotation among team members: compromised follow-up
- Drops out and missing data
- Watch running out of battery and recording problems influence on measures collection
- Some data extracted from the activity tracker (step count)

**Opportunities**

- Primary care setting
- Loan of an activity tracker and iPad: money saved
- Common technology

**Improvements Opportunities-Strengths**

- Integration of the activity tracker data in the electronic medical record: physicians and health professional teams could get objective information on physical activity
- Organization of academic health centers to facilitate the contribution of physicians and other health professionals in research
- Follow-up for a longer period, for example, 6 months

**Improvements Opportunities-Weaknesses**

- Larger study with more participants
- Extraction of all the activity tracker data
- Collection of pre- and postintervention data to truly compare both groups
- Clarification of objectives at the beginning of the study
- Formation of groups for motivation

**Threats**

- Recruitment (1 year)
- Coordination and synchronization, too many professionals from different establishments
- Less-than-optimal communication between professionals
- One academic health center
- Technology should be available to all
- Difficulty synchronizing the activity tracker and app
- Technology is harder to use for certain people
- Watch: lack of batteries
- Extraction of the activity tracker data difficult, forgotten login access

**Improvements Threats-Strengths**

- Team 100% dedicated to the clinical intervention and in the same establishment
- All team members available during a time slot
- Addition of more academic health centers or clinical establishments
- Limit on the number of professionals involved (one professional does the tests)

**Improvements Threats-Weaknesses**

- Full-time research coordinator dedicated to the study (follow-up and recruitment), paid by the state, worked in the academic health center with health professionals
- Scientific counselor for issues such as optimizing the recruitment process
- Training on how to use the device and a person to join if further help is needed
- Team being immediately informed when a technological problem occurs to avoid data loss
- Logging access given by the team

### Strengths

#### Study Design and Team

The project’s initiation by a patient partner who was involved throughout the study, from the beginning to the end, was considered a major strength. One team member mentioned:

The initial idea of the research project was proposed by a patient partner. The patient partner wanted to follow the physical activity with an activity tracker in order to discuss daily data with the health care team and optimize the management of his chronic disease. It demonstrates that this research answered both patients’ needs and researchers’ clinical questions. The layperson participated in all the meetings to organize and operationalize the study. From the beginning of the study, the patient partner gave his point of view on the study design, activity tracker measurements, satisfaction and implementation questionnaires, development of the SWOT matrix, barriers and facilitators and knowledge transfer strategies such as presentation of the results at scientific congresses, contribution to the scientific article as co-author, etc. An interesting point to note is that we had the layperson’s suggestions and commentaries quickly and this was helpful to optimize the feasibility of the study in primary care and compliance with the device in a real-life setting. The co-construction of the research project with a patient partner was meaningful and a rewarding experience.

Team member 1

The collaboration of many health professionals from the scientific field also optimized knowledge transfer and allowed a better understanding of research in primary care, according to the team members. Some characteristics of the team members were mentioned, including, “The presence of a skilled research professional ensuring the participant’s follow-up, the leadership of the clinician-researcher in charge, the accessibility of the principal investigator” [Team member 2].

The follow-up with the kinesiologist was considered a positive aspect of the study design as it encouraged patients and “optimized the use of the activity tracker (goals and PA intensity)” [Team member 3].

#### Device

The high level of participants’ satisfaction regarding the device was perceived as a major strength. The motivational aspect of the device was also mentioned: (1) “The use of a technological tool to track more objectively PA was appreciated by patients. Actually, there are few motivational tools used by physicians to track PA” [Team member 1], and (2) “The possibility with technology to motivate ourselves to initiate healthy lifestyle habits and maintain them” [Team member 4].

### Weaknesses

#### Budgetary Constraints and Implications

One of the main weaknesses in the study was the small budget, which limited the number of pedometers and activity trackers and subsequently the sample size (30 patients). The part-time research coordinator, due to the limited budget, may have had an impact on the follow-up on measures and missing data:

Part-time research coordinator: I think, for an intervention study on lifestyle habits in primary care, adding a technological device implies a follow-up on many measures with the collaboration of different professionals, a full-time research coordinator is a must. Research coordinators help investigators conduct clinical trial and play an important role to foster the collaboration between clinicians and research team. Indeed, if a patient needs help for technological issues, it is easier to reach the research coordinator when available full-time.

Team member 1

#### Turnover and Technical Issues

Staff turnover due to uncontrollable reasons had a major impact on the follow-up on data extraction, cardiometabolic risk measurements, and research trajectory and was therefore perceived as a weakness. Due to technical problems during data download, the data from a few patients’ devices could not be extracted: 4 participants had missing data, 1 had withdrawn, and there was a problem extracting data for 3 participants who forgot their password.

### Opportunities

#### Primary Care Setting

The primary care setting was seen by the team as the principal opportunity. The study took place in a primary care setting in an academic health center, which allowed research students to be trained and introduced primary care research to health professionals, clinicians, and patients. For instance, a medical student completed her master’s degree with this project. Results were presented at provincial, national, and interventional congresses, and an article was published in a scientific journal. Thus, this research optimized faster knowledge transfer from research to the medical setting.

#### Loan of Equipment

The activity tracker and iPad were loaned by the researcher, who is specialized in IT (Canada Foundation for Innovation). This collaboration combined both expertise and resources. There was no donation from the manufacturer or sponsorship.

#### Common Technology

The fact that an activity tracker is a common technology available to the public is also a factor contributing to the real-life setting. Fitness trackers are readily available at a relatively affordable cost and are not exclusive to research. It is an increasingly popular and growing technology. Given that it is a common technology, the outcomes of interdisciplinary team care for the patient can include using mobile apps that track lifestyle change progress and that prompt lifestyle intervention. Support with common digital technology (eg, apps, wearable devices) is a key construct for effective, sustainable patient care self-management.

### Threats

#### Recruitment Issues

The difficulty of recruiting participants from the medical clinic was perceived as the biggest threat by the research team:

Surprisingly, the recruitment of diabetic patients being sedentary remained a big challenge even if the study took place in a primary care setting and a clinic with many of them. Patients were interested to participate in the research project, but we had few references from the nurses and physicians. Despite the reminders to the team of health professionals, the recruitment took about one year.

Team member 1

#### Administrative Challenges

The team felt it was a challenge to coordinate the necessary resources for recruitment and participants’ follow-up:

Turnover among professionals who contributed to the intervention with participants was a challenge. This meant there were many steps to plan, which sometimes seemed heavy for the participants and the professionals involved. […] The professionals in the project did not all come from the same organization, which complicated the logistics […] The communication between professionals was not always adequate either, which led to confusion in the participants’ follow-up.

Team member 5

#### Technological Difficulties

The difficulty synchronizing the activity tracker and app was mentioned by the team, as was the watch’s lack of batteries. The team also added, “The use of an activity tracker can be challenging for certain people, including the elderly” [Team member 3]. Some patients forgot their password, which caused problems with data extraction as well as watch battery running out and recording problems that influenced measure collection.

#### Single Research Site

The study was limited to one research site and participants with diabetes. As well, there was only one family medicine unit included in the study. One member also mentioned equipment availability as a concern: “We have to find a way to make this technology available for all” [Team member 4]. Thus, the possibility of conducting a multicenter trial was limited.

### Improvements

The improvements suggested by the research team and the participants were classified according to the O-S, O-W, T-S, and T-W strategies (Textbox 1). The main points for O-S were (1) integration of activity tracker data in the electronic medical record, (2) organization of academic health centers, and (3) follow-up on a longer period. The main points for O-W were (1) a larger study with more participants; (2) extraction of all the activity tracker data; (3) collection of data in pre-post intervention, clarification of objectives; and (4) formation of groups of motivation. The main points for T-S were (1) full-time team dedicated and available to the clinical intervention, (2) addition of more academic health centers, and (3) limit the number of professionals involved. The main points for T-W were (1) full-time research coordinator, (2) scientific counselor, (3) training on how to use the device, (4) team training with the technological tool, and (5) logging access given by the team.

### Feasibility and Implementation

Overall, the team members felt that the implementation in primary care was feasible:

There should be a way that the relevant activity tracker data can be integrated into the electronic medical record so physicians can access them and discuss with the patient. They could have a more objective approach to tracking PA […] and then establish a treatment plan and refer to the appropriate health professionals.

Team member 1

These devices’ effects have been demonstrated; it is time these trackers can be prescribed and reimbursed.

Team member 2

Another team member mentioned that “it [activity tracker] brings objective data on PA and could improve physician-patient communication” [Team member 3]. One team member felt some people might benefit more from it: “I think that this device is interesting mostly in cases of instability or huge variability in results/medical analyses of patients. The device allows us to see fluctuations and potentially establish certain tendencies” [Team member 5].

## Discussion

### Principal Findings

This is a 3-month pilot study conducted in Quebec, Canada, that aimed to assess the satisfaction of patients with type 2 diabetes who use an activity tracker to increase motivation for PA, as well as to investigate the patients’ and team's perception of the implementation of this technology in a primary care facility. According to this research, patients with type 2 diabetes were satisfied with their activity tracker, which was used to increase PA motivation. Members of the health care and research teams agreed that this novel technology tool can be used in primary care, although there are still obstacles to its frequent use in clinical practice.

### Implementation and SWOT Analysis

#### Satisfaction and Acceptability: Patients’ Perspective

Most of the participants were satisfied with the use of their activity tracker and with the technical support provided by the research team. They found the information useful, with the step count being perceived as the most useful parameter to track PA and for PA motivation. Overall, participants reported an increase in their PA and all of them did stick to their PA program once the study was over, with the activity tracker playing a substantial role. One Canadian out of 4 (24%) owns at least 1 connected device allowing health or wealth data capture, of which 88% of them have a smart watch or bracelet [23]. Ware et al [24] explored Canadian older adults’ perceptions of the use of eHealth technologies. Their findings support the potential value they perceive in eHealth technologies, particularly in their ability to give access to personal health information and facilitate communication between providers and peers living with similar conditions. We consider that in our study this technology was well accepted by older individuals (the mean age of participants was 62 years), as demonstrated by the high satisfaction with the device used. Ummels et al [10] described the experience of commercially available activity trackers embedded in the physical therapy of patients with a chronic disease. Participants perceived the activity tracker as a motivation to be more physically active and reach their goals, similar to our findings [10]. However, participants experienced some technical failures too and found it complex [10].

#### Health Care Team Members’ Perspective

According to the team members’ perceptions, the main strengths were the project initiation and involvement of a patient partner, the study design and team, as well as the device used. Patients’ opinions during the course of the study in a real-world context gave credibility to the study and increased the research quality. Patient engagement in health research is an emerging phenomenon and contributes, among others, to identify research questions and outcomes important to patients and clinicians, data collection processes, interpretation of results, and dissemination [25,26]. Indeed, patient engagement allows patients to become partners with academic researchers to create a meaningful and active collaboration in governance, priority setting, conducting research, and knowledge transfer [27,28]. Patient engagement helps transfer research findings into practice and can ultimately improve patients’ outcomes [29]. Furthermore, patient-centered design for digital health facilitates implementation and improves the relevance of research and its uptake into health care [30]. Another interesting point to discuss is that our study was designed as a mixed methods study, which could represent a strength. Both quantitative and qualitative data (from randomized controlled trial participants) were collected by using a questionnaire. While the qualitative data was obtained from the questionnaire in the intervention group, this design can dilute the strengths of both methods. However, the questionnaire for the team members and participants was based on the validated models analysis [15-18]. A suggested solution will be to extend the study with more patients and to recommend the qualitative design as the main method for the participants (eg, in-depth interviews). The hybrid type 1 design itself, combining dual testing such as a randomized pilot trial and SWOT analysis, allowed us to collect valuable information for use in subsequent implementation research trials (hybrid or not). All the information gathered in our matrix will help speed the translation of our research findings into routine practice, develop more effective implementation strategies, and provide more useful information for decision makers.

The principal weaknesses were the budgetary constraints, the turnover, and the technical issues. The budgetary constraints had an impact on sample size, duration and design of the study, cardiometabolic measurement choices, number of pedometers and activity trackers, human resources, and so on. The turnover compromised the follow-up and consequently had an impact on technological failure. The technical issues were missing data, recording problems, and a lack of data extraction, as previously reported in other studies, and were related to turnover of health professionals and the part-time research coordinator [3,4,9-11].

The opportunities were the real-life setting in a primary care setting and the common technology. Primary care and primary care academics have steadily contributed to many aspects of health research, but they have been particularly important in applied research at the structural and inspirational levels [31]. With the increasing use of digital solutions, there is a growing need to evaluate their impact in primary care, including risks and benefits, and to inform health policies that are both patient-centered and evidence-based [32]. The authors have proposed 5 wishes for the future of digital care, such as co-design with primary health care professionals and patients, better infrastructures, support and training, data sharing, clear regulations and best practice standards, and ensuring patient safety and privacy [32]. The COVID-19 pandemic has shown the need and relevance of collaboration as part of a global community to develop a shared agenda that supports collaboration in general practice, research, and policy, and facilitates the delivery of digital solutions that leave no one behind [32]. Support with common digital technology (eg, apps and wearable devices) is a key construct for effective, sustainable patient care self-management. Two studies in type 2 diabetes patients with activity trackers show maximum effects at the beginning of the study, within 2 months. [2,33] Thus, such renting or lending could be beneficial, and those who really enjoyed their experience could actually buy an activity tracker on their own afterward, just like these 55% (6/11) planned to do after this study.

The main threats were the recruitment issues, the administrative challenges, the technological difficulties, and the single research site. The administrative challenges were the recruitment of patients lasting 1-year, the coordination or synchronization of too many professionals from different establishments, and less-than-optimal communication between professionals. The technological difficulties were: difficulty synchronizing the activity tracker and application, technology harder to use for certain people, extraction of the activity tracker data, forgotten login access, and so on. There is a need to develop patient recruitment strategies that minimize the efforts required by staff to recruit patients while meeting privacy and ethical responsibilities and minimizing the risk of selection bias, as studies have identified barriers to the recruitment of patients in a primary care cluster randomized trials [34]. Another exploratory study provides preliminary evidence of an internal structure to optimize recruitment in primary care [35]. In the fall and winter, the recruitment was more difficult as participants were less willing to practice PA outdoors due to the weather. Since participants in the study knew they were being observed, it was possible that they changed some of their habits.

### Challenges and Strategies of Implementation

Improvements included the integration of relevant activity tracker data in the electronic medical record, involving more family medicine units, repeating the study with more participants, and hiring a full-time research coordinator. Most of the team members perceived that an activity tracker could be integrated into the follow-up of patients in primary care. Overall, participants appreciated the activity tracker, as did the team, but as shown by the SWOT analysis, there are some challenges. The results obtained are consistent with the preliminary literature regarding the implementation of activity trackers in primary care. Reed et al [9] reported an increase in PA among inactive rural adults when implementing a 12-week Fitbit-based intervention and technological difficulties, while the nursing staff cited human resources and money as barriers. Similar work has been completed among both patients who had diabetes earlier [36] and those with diabetes and other cardiometabolic conditions reported in a recent systematic review and meta-analysis [37].

We believe that to facilitate digital health research in primary care, it is essential to provide a structure dedicated to supporting clinical research using digital technologies, which requires an adequate organization of academic health centers, substantive funding, and human resources. The clinical research coordinator supports and oversees the daily activities and plays a critical role in the conduct of the clinical trials. It is suggested that a research coordinator should be hired in academic health centers to optimize clinical research in the primary care setting.

The next step is to integrate the activity tracker into the electronic medical record, as suggested by our team. Shannahan et al [38] demonstrated that the patients’ activity tracker data can be embedded within visits with primary providers to personalize recommendations and that patient-physician information sharing is feasible. They conclude that activity trackers may foster patient-physician communication regarding PA, but infrastructure and resources are needed [38]. Bliudzius et al [39] stated that the data from physical monitoring systems and external medical devices should be integrated into the medical record system as these are essential in clinical work. Moreover, system integration is useful to make detailed analyses and have a global or clear picture of the patient’s health [39]. We strongly believe that patient PA data and other cardiometabolic parameters are essential in clinical work, especially in primary care settings. It helps health care practitioners to review and use patient data collected, understand how the patient feels in real-life situations, adhere to the physician’s or health professional team’s recommendations, and thus solve problems faster [39]. In order to implement the technology on a larger scale, it should be important to obtain consent and explore the patients’ stress over having their data shared with a third party. As reported by Hodgson et al [4], there is future scope for using Fitbit activity trackers to support active lifestyles in adults diagnosed with type 2 diabetes. More detailed discussion with health care professionals could identify methods of integrating activity trackers into the care of patients.

### Limitations of the Study Design

This study has a number of limitations. The first limitation is the small sample size, which could compromise the power of the study. However, the sample size was determined for the participant according to the availability of the equipment, and for the team members, it was determined according to their involvement in the pilot study. We believe that we have gathered sufficient helpful information to draw general recommendations to optimize the implementation of this technological tool in primary care. It is also important to report that some satisfaction may be tracker-dependent. The use of more sophisticated devices (eg, AppleWatch) may increase the satisfaction but also cause some other technical issues (eg, the download of the data). Therefore, some conclusions should be reformulated as “device-related,” since changing the used device may give different results. Another limitation is the relatively short-term follow-up period of 3 months, which was selected in the context of a pilot study to prevent an increased withdrawal rate in the control group and to align with our budget. However, we are aware that a longer duration may have been beneficial for incorporating lifestyle habits and also for a long-term comparison with the control group. Moreover, a longer duration could be interesting to observe the tendency of motivation over time because the highest step average was recorded after the follow-up phone call and the lowest weekly step average was recorded at week 12 at the end of the intervention. A long-term study will be interesting to observe the motivation tendency and find solutions to optimize and maintain PA motivation with an activity tracker over time. It is interesting to point out that most studies, even pilot studies, have recommended a follow-up duration of at least 6 months and preferably 12 months or longer. Similar studies had a follow-up period of 3 to 6 months [1,2,40]. A meta-analysis of activity trackers in adults with cardiometabolic conditions reports a median duration of 17 weeks and up to 18 months [37]. Another study on young adult cancer survivors found that a 12-week Fitbit and Facebook-based physical intervention was feasible for this population and had promising effects on reducing sedentary time [41].

### Conclusions

In conclusion, patients with type 2 diabetes were satisfied with the activity tracker and felt that it incited them to stick with their PA program once the study was over. According to the research team, its implementation is feasible in primary care, but some challenges remain to having this technological tool in clinical practice. From a clinical innovation perspective, it would be interesting to find a way to synchronize relevant activity tracker data to the electronic medical record to optimize the collaboration between patients and health professionals in a primary care facility.

Based on a patient partner’s idea and his continuous involvement, this project showed that laypersons have an important role in implementation research by informing the design of realistic interventions and optimizing their feasibility. Moreover, health researchers, clinicians, and other health care professionals need to clarify the opportunities to integrate digital technologies into public health to maximize their potential to improve public health outcomes and patient care.

## Appendix

Multimedia Appendix 1 Satisfaction and Acceptability of the Activity Tracker: Patients' Feedback.

Multimedia Appendix 2 Implementation Questionnaire: Health Care Team's Feedback.

## Abbreviations

O-S: Opportunities-Strengths
O-W: Opportunities-Weaknesses
PA: physical activity
SWOT: strengths, weaknesses, opportunities, and threats
T-S: Threats-Strengths
T-W: Threats-Weaknesses

## Data Availability

Questionnaires are available in the supplementary files. The data sets used and analyzed during this study are available from the corresponding author on reasonable request.
