# Supplementary material for: Implementing an Activity Tracker to Increase Motivation for Physical Activity in Patients With Diabetes in Primary Care: Strengths, Weaknesses, Opportunities and Threats (SWOT) Analysis
Source: JMIR Form Res. 2023 Mar 10;7:e44254. doi: 10.2196/44254 (PMC10039411; doi:10.2196/44254)
Supplement: Multimedia Appendix 1 [file formative_v7i1e44254_app1.pdf]

Questionnaire of satisfaction and acceptability for the activity tracker (Fitbit watch) and its application (English translation of the original French questionnaire) inspired by the Technology Acceptance Model

1) How satisfied are you with the use of the activity tracker that you had to wear in the last 3 months?

1) Not at all satisfied

2) Not very satisfied

3) Somewhat satisfied

4) Satisfied

5) Very satisfied

2) How satisfied are you with the technical support provided for the use of the activity tracker and its application on the Ipad?

1) Not at all satisfied

2) Not very satisfied

3) Somewhat satisfied

4) Satisfied

5) Very satisfied

3) How would you describe the information displayed by the activity tracker and its application on the Ipad according to the objectives you had targeted with the kinesiologist?

____

4) To what extent did you find the information provided by the device and its application useful?

1) Not at all useful

2) Not very useful

3) Somewhat useful

4) Useful

5) Very useful

5) What is the principal change you made to your lifestyle habits during the study?

____

6) Which parameter (s) do you think would be most useful for tracking your physical activity?

1) Weight

2) Distances traveled

3) Step count

4) Minutes of being sedentary, mild, moderate, and strenuous physical activity

5) Calories burned

7) Which parameter (s) do you think is the most useful to motivate you to be physically active?

1) Weight

2) Distances traveled

3) Step count

4) Minutes of being sedentary, mild, moderate, and strenuous physical activity

5) Calories burned

8) Did you continue to integrate physical activity into your daily routine after the study and why?

If yes :

a) **To which extent has the activity tracker incited you to continue your physical activity program once the study was over?**

1) Not incited at all

2) Not very incited

3) Somewhat incited

4) Incited

5) Very incited

If no :

b) **In the event that your lifestyle has not changed, please explain why:**

____

9) Do you plan to purchase an activity tracker after the study?

____

10) Do you have feedback you would like to share with us regarding the study and its progress?

____

References

Davis FD. Perceived Usefulness, Perceived Ease of Use, and User Acceptance of Information Technology. MIS Quarterly. 1989;13(3):319-40.

Brooke J. SUS: A quick and dirty usability scale. Usability Evaluation in Industry. CRC Press; 1996. p. 189-94.

Pande T, Saravu K, Temesgen Z, Seyoum A, Rai S, Rao R, et al. Evaluating clinicians' user experience and acceptability of LearnTB , a smartphone application for tuberculosis in India. mHealth. 2017;3(7). PMID: 28828377
