# Supplementary material for: Implementing an Activity Tracker to Increase Motivation for Physical Activity in Patients With Diabetes in Primary Care: Strengths, Weaknesses, Opportunities and Threats (SWOT) Analysis
Source: JMIR Form Res. 2023 Mar 10;7:e44254. doi: 10.2196/44254 (PMC10039411; doi:10.2196/44254)
Supplement: Multimedia Appendix 2 [file formative_v7i1e44254_app2.docx]

**LimeSurvey questionnaire: Evaluation of the Implementation of an Activity Tracker (Fitbit) by SWOT Analysis (English translation of the original French questionnaire)**

The **SWOT** (Strengths, Weaknesses, Opportunities and Threats) **analysis** are assessed against a plan, project or organization [15]. Strengths and weaknesses are part of the internal analysis which includes resources, capabilities, key competencies and competitive advantages [15]. Rather, opportunities and threats are components of external analysis, which includes the resources of competitors, the industrial environment as well the broader environment (political, ecological, sociocultural, ethical, legislative, etc.) [15]. The purpose of a SWOT analysis is to use knowledge of strength, weaknesses, opportunities and threats to formulate future strategies [15]. More specifically, the purpose of this questionnaire is to assess the components of the SWOT analysis related to the implementation of an activity tracker (Fitbit) in a diabetic population in a primary care setting.

References :

[15]. Sammut-Bonnici T, Galea D. Volume 12. Strategic Management: SWOT analysis. In: Wiley Encyclopedia of Management. Hoboken, New Jersey: John Wiley & Sons, Inc; 2015.

There are 11 questions in this questionnaire related to the SWOT analysis.

1) How old are you?

_________ years

2) How would you describe yourself?

Please select only one of the following:

1. Man
2. Women
3. Another identity

3) What is your role in the project?

Please select only one of the following:

1. Principal investigator
2. Co-investigator
3. Patient
4. Patient partner
5. Kinesiologist
6. Research assistant

4) Does the project have any **strengths**? If so, what are they? Please write your answer below:

____________________________________________________________________________________________________________________________________________________________

5) Does the project have any **weaknesses**? If so, what are they? Please write your answer below:

____________________________________________________________________________________________________________________________________________________________

6) Knowing that the first objective of the study was to assess the feasibility of implementing an activity tracker (Fitbit Charge HR watch) in primary care, has this objective been achieved? Why?

Please write your answer below:

____________________________________________________________________________________________________________________________________________________________

7) Knowing that the second objective was to assess the impact of an activity tracker on physical activity and cardiometabolic risk variables, has this objective been achieved? Why?

Please write your answer below:

______________________________________________________________________________

______________________________________________________________________________

8) Did you encounter any **barrier** (s) and/or element (s) that were **harmful/threatful** during the intervention? Which component (s)?

Please write your answer below:

______________________________________________________________________________

______________________________________________________________________________

9) Is/are there any component (s) that facilitated the intervention in primary care and that could be taken as an **opportunity**? Which component (s)?

Please write your answer below:

______________________________________________________________________________

______________________________________________________________________________

10) Do you think an activity tracker such as the one used in this study could be integrated into the follow-up of patients in primary care? Why?

Please write your answer below:

______________________________________________________________________________

______________________________________________________________________________

11) What could be improved in a future intervention to optimize the implantation of a similar technological device in a primary care setting?

Please write your answer below:

______________________________________________________________________________

______________________________________________________________________________

Submit your questionnaire

Thank you for completing this questionnaire.
